# Risk reduction of contralateral breast cancer and survival after contralateral prophylactic mastectomy in *BRCA1* or *BRCA2* mutation carriers

**DOI:** 10.1038/sj.bjc.6602703

**Published:** 2005-07-19

**Authors:** T C van Sprundel, M K Schmidt, M A Rookus, R Brohet, C J van Asperen, E J Th Rutgers, L J van‘t Veer, R A E M Tollenaar

**Affiliations:** 1 Department of Surgery, D6-44, Leiden University Medical Center, PO Box 9600, 2300 RC Leiden, The Netherlands; 2 Department of Pathology, The Netherlands Cancer Institute/Antoni van Leeuwenhoek Hospital, Amsterdam, The Netherlands; 3 Department of Epidemiology, The Netherlands Cancer Institute/Antoni van Leeuwenhoek Hospital, Amsterdam, The Netherlands; 4 Department of Clinical Genetics, Leiden University Medical Center, Leiden, The Netherlands; 5 Department of Surgery, The Netherlands Cancer Institute/Antoni van Leeuwenhoek Hospital, Amsterdam, The Netherlands

**Keywords:** breast cancer, *BRCA1*, *BRCA2*, mastectomy, surgery, prevention

## Abstract

The clinical outcome of contralateral prophylactic mastectomy (CPM) in women with a *BRCA1* or *BRCA2* mutation and a personal history of invasive breast cancer is unknown. We identified a cohort of 148 female *BRCA1* or *BRCA2* mutation carriers (115 and 33, respectively) who previously were treated for unilateral invasive breast cancer stages I–IIIa. In all, 79 women underwent a CPM, while the other women remained under intensive surveillance. The mean follow-up was 3.5 years and started at the time of CPM or at the date of mutation testing, whichever came last, that is, on average 5 years after diagnosis of the first breast cancer. One woman developed an invasive contralateral primary breast cancer after CPM, whereas six were observed in the surveillance group (*P*<0.001). Contralateral prophylactic mastectomy reduced the risk of contralateral breast cancer by 91%, independent of the effect of bilateral prophylactic oophorectomy (BPO). At 5 years follow-up, overall survival was 94% for the CPM group *vs* 77% for the surveillance group (*P*=0.03), but this was unexpectedly mostly due to higher mortality related with first breast cancer and ovarian cancer in the surveillance group. After adjustment for BPO in a multivariate Cox analysis, the CPM effect on overall survival was no longer significant. Our data show that CPM markedly reduces the risk of contralateral breast cancer among *BRCA1* or *BRCA2* mutation carriers with a history of breast cancer. Longer follow-up is needed to study the impact of CPM on contralateral breast cancer-specific survival. The choice for CPM is highly correlated with that for BPO, while only BPO leads to a significant improvement in overall survival so far.

Women identified as carriers of a mutation in one of the breast and ovarian cancer-susceptibility genes *BRCA1* or *BRCA2* have strongly elevated risks of developing breast or ovarian cancer (Ford *et al*, 1998). A recent meta-analysis (Antoniou *et al*, 2003) including 22 studies, revealed an average cumulative risk of 65% for breast cancer and 39% for ovarian cancer in *BRCA1* mutation carriers by age 70 years. The corresponding estimates for women with a mutation in *BRCA2* were 45 and 11%. Once diagnosed with breast cancer, these women are also at high risk of developing breast cancer in the contralateral breast. Early reports of The Breast Cancer Linkage Consortium estimated a contralateral breast cancer cumulative risk of 50–60% at age 70 years in *BRCA1* or *BRCA2* mutation carriers (Easton *et al*, 1995; The Breast Cancer Linkage Consortium, 1999). Later studies estimated even higher incidences of contralateral breast cancer within the first 5 years of follow-up after the primary breast cancer: 12–33% among *BRCA1* or *BRCA2* mutation carriers (2.4–6.5% per year) (Robson *et al*, 1998; Verhoog *et al*, 1998, 1999) as compared to a 0.4–1% per year for breast cancer patients in general (Fisher *et al*, 1984).

Owing to the elevated risks and fear of contralateral breast cancer, some women opt for contralateral prophylactic mastectomy (CPM). No studies to date exist on the efficacy of CPM in carriers of *BRCA1* or *BRCA2* mutations. Two retrospective studies have evaluated a heterogenic population of breast cancer patients with variable extent of family history and reported a reduction of contralateral breast cancer after CPM, but without improvement of overall survival in a follow-up period of 7–10 years (Peralta *et al*, 2000; McDonnell *et al*, 2001). A recent systematic review included three additional studies; however, these lacked a comparison group and/or follow-up was not standardised (Lostumbo *et al*, 2004). Therefore, in the present study, the efficacy of CPM was investigated in a group of women with pathogenic *BRCA1* or *BRCA2* mutations and a personal history of unilateral invasive breast cancer.

## PATIENTS AND METHODS

### Identification of patients

All patients identified until June 2003 as being a *BRCA1* or *BRCA2* mutation carrier, with previous history of unilateral, stage I–IIIa, invasive breast cancer, from The Netherlands Cancer Institute, Amsterdam (*n*=101) and The Leiden University Medical Centre, Leiden (*n*=47), were included. All women underwent surgery for their primary breast cancer, either breast-conserving surgery or mastectomy. In addition, all women were counselled and monitored at the Family Cancer Clinic and outpatient clinic at each institute. *BRCA1* or *BRCA2* mutation status was determined by direct mutation testing. Women with so-called unclassified variants were not included in this study. Data on mutation status, date of disclosure DNA test result, pathological features and treatment of the primary breast cancer as well as recurrence, bilateral (prophylactic) (salpingo-)oophorectomy status (BPO), other carcinomas, last date of follow-up and vital status were extracted from medical files, operation and pathology reports.

### Surgical techniques and surveillance

CPM was defined as surgical removal of the opposite breast in a patient with previous unilateral invasive breast cancer, provided that no suspicion for cancer in the opposite breast was apparent by means of preoperative physical, radiological and, if applicable, pathological examination. In case of a CPM, either a skin sparing or a simple total mastectomy was performed. In women who previously had undergone breast-conserving therapy for their first breast cancer, a residual mastectomy procedure was carried out on the ipsilateral breast at the time of CPM. In all cases, the nipple–areolar complex was removed, while the pectoralis muscles were preserved, except its fascia. Axillary node dissection was not performed. However, in case of accidental removal of some superficial axillary lymph nodes, these were examined histologically. After CPM, women returned yearly for a physical examination.

All CPM breast specimens were postoperatively examined for lesions by experienced pathologists at each institution. Three ductal carcinomas *in situ* and one 3.2 cm large, invasive ductal carcinoma Bloom & Richardson nuclear grade III were detected in the specimens obtained at the time of prophylactic mastectomy. Preoperative clinical or radiological assessment had not revealed abnormalities.

According to Dutch guidelines, women with a *BRCA1* or *BRCA2* mutation are enrolled in the surveillance-screening program from age 25 years. This is continued after a first breast cancer for remaining breast tissue and the opposite breast. Regular surveillance consisted of a monthly breast self-examination, semiannually clinical breast examination and yearly mammography.

### Statistical analysis

We addressed two main questions, first whether risk of contralateral breast cancer was different between the surveillance and CPM group, and second whether (contralateral) breast cancer specific and overall survival differed between the groups. The start of follow-up and inclusion criteria for analysis were chosen such that the risk reduction of CPM for contralateral breast cancer would not be overestimated (Klaren *et al*, 2003). Hence, the start of follow-up was defined in the surveillance group as date of mutation testing and in the CPM group as date of mutation testing or CPM, whichever came last. In the surveillance group, mutation testing was always performed after diagnosis of the first breast cancer. In the CPM group, 15 patients were tested before diagnosis of their first breast cancer; however, by definition the date of CPM was taken as the start of follow-up. Only in 11 patients, the results of mutation analysis became available after their CPM.

In the contralateral breast cancer risk analysis, patients in the surveillance group who experienced contralateral breast cancer before the date of mutation testing (*n*=26) were excluded, as well as four patients in the CPM group in whom *in situ* or invasive carcinoma was detected at the time of prophylactic mastectomy. Patient and treatment characteristics did not significantly differ between patients who were kept in the contralateral breast cancer risk analysis (*n*=118 out of 148) and patients who were excluded (*n*=30), except that the latter had received less often chemotherapy (13.3 *vs* 53.5%, *P*=0.001), had lower stage (53.3 *vs* 27.9% stage I, *P*=0.049), were diagnosed in earlier years (median 1990 *vs* 1994, *P*=0.001) and had a longer delay between primary breast cancer and date of mutation analysis (9.3 *vs* 5.3 years, *P*=0.003). The four patients excluded from the CPM group were all still alive. Of the 26 patients, 24 excluded from the surveillance group were still alive, one died of breast cancer and one died of ovarian cancer.

In the survival analysis, all patients, also the 26 patients with contralateral breast cancer before the date of testing, were included.

*χ*^2^ tests, one-way ANOVA, Mann–Whitney *U*-tests and *t*-tests were used to compare the patient characteristics. Kaplan–Meier curves with log-rank test and Cox's regression models were used to evaluate the incidence of contralateral breast cancer and overall or breast cancer-specific survival. In the Cox's regression models, time between first breast cancer and start of follow-up (to adjust for survival bias), year of diagnosis first breast cancer, age at mutation analysis, age at diagnosis first breast cancer, BPO, surgery (breast-conserving surgery or mastectomy), chemotherapy, radiotherapy and stage of first breast cancer were evaluated as confounders. If the univariate estimate of the hazard ratio (HR) for CPM changed more than 10% if a potential confounder was added to the model, this factor was accepted as a confounder. A two-sided *P*-value of <0.05 was considered to indicate statistical significance. All analyses were performed using SPSS Release 10.07 (Chicago, IL, USA).

## RESULTS

### Patient characteristics

The clinicopathological characteristics of the 148 women at time of the primary breast cancer diagnosis are summarised in Table 1. Eventually, 79 women opted for a CPM and 69 women remained under close surveillance. The calendar year of first breast cancer diagnosis was significantly later in the CPM group than in the surveillance group, that is, 4 years.

The patient characteristics at the time of CPM and of women under surveillance are listed in Table 2. The gene mutations in *BRCA1* or *BRCA2* were equally distributed between the two groups. The mean age at CPM was close to the mean age at mutation analysis for this group with an average interval between the first breast cancer diagnosis and CPM of 3.9 years, while women in the surveillance group were on average tested at an older age. Women with a CPM underwent more often an oophorectomy, especially if considering BPO, and were significantly younger when they underwent this surgical intervention than women in the surveillance group. Considering the period from diagnosis of first breast cancer until the end of follow-up (10.5 years in the surveillance group *vs* 7.4 years in the CPM group), in the complete surveillance group 32 contralateral breast cancers were observed, as compared to only one in the CPM group (Table 3a). Recurrence of breast cancer did not differ between the CPM and surveillance group (Table 3a).

### Risk of contralateral breast cancer

For the analyses of contralateral breast cancer, follow-up started at the date of mutation testing or the date of CPM, whatever came last (see also Statistical analysis). The mean follow-up did not differ markedly between both groups, 3.4 *vs* 3.1 years, respectively (Table 3b). Six women (14%) in the surveillance group developed contralateral breast cancer after a mean of 2.2 years follow-up (mean interval 9.0±6.2 years after the first breast cancer diagnosis). The one case of contralateral breast cancer observed in the CPM group occurred in a 62-year-old woman 1.6 years after the start of follow-up, and 5.4 years after the first breast cancer diagnosis. A 1.8 cm large, invasive ductal carcinoma grade III (Bloom & Richardson) was detected in minimal residual mammary gland tissue. At the end of the follow-up period, this woman was still alive and had no evidence of disease.

**Figure 1:**
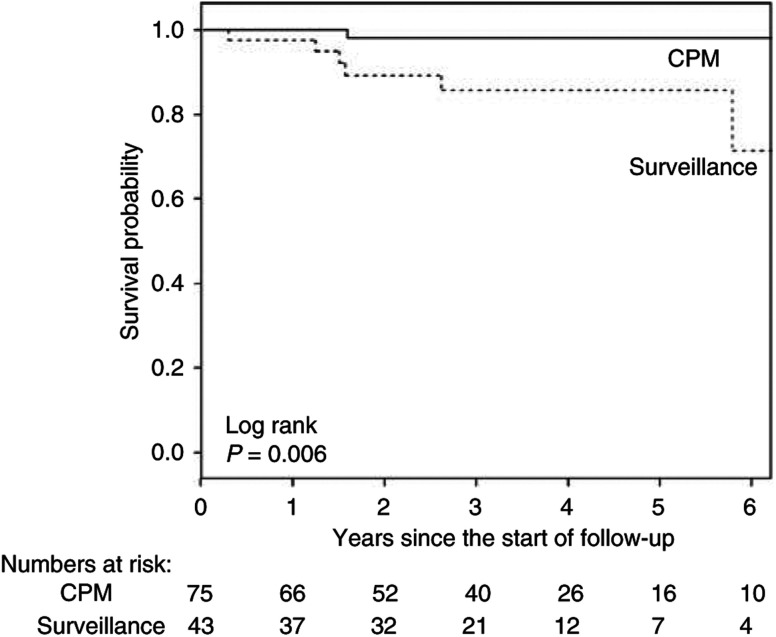
Contralateral breast cancer-free survival in patients who opted for CPM *vs* patients who remained under surveillance.

The contralateral breast cancer-free survival in the CPM group was significantly lower as compared to the women under surveillance (log rank, *P*=0.006) (Figure 1). Cox's proportional-hazards analysis showed that CPM significantly (*P*=0.028) decreased the risk of contralateral breast cancer (HR 0.09 (95% confidence interval (CI), 0.01–0.78)) (Table 4). Also within the group of women with a BPO, the risk of contralateral breast cancer was decreased after a CPM (3 *vs* 1 contralateral breast cancer in 39 women under surveillance and 61 women with CPM, respectively).

### Breast cancer-specific and overall survival

**Figure 2:**
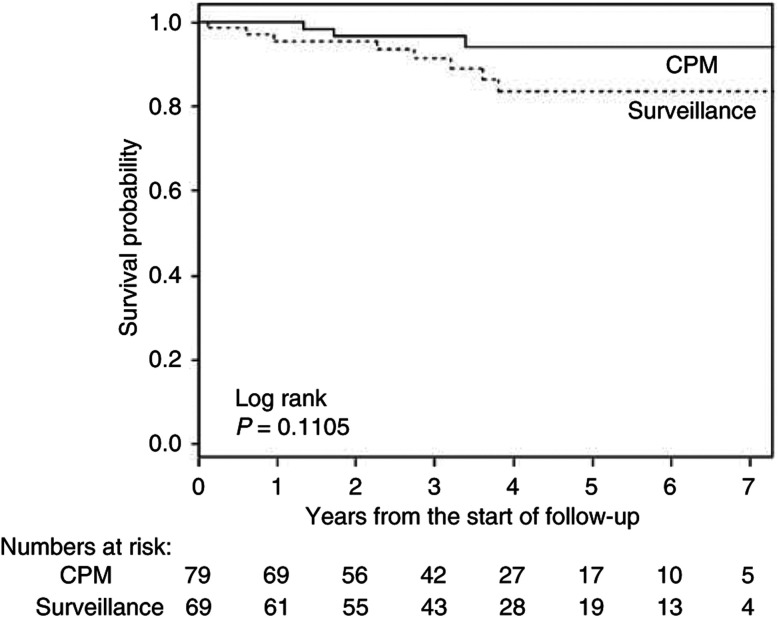
Breast cancer-specific survival in patients who opted for CPM *vs* patients who remained under surveillance.

**Figure 3:**
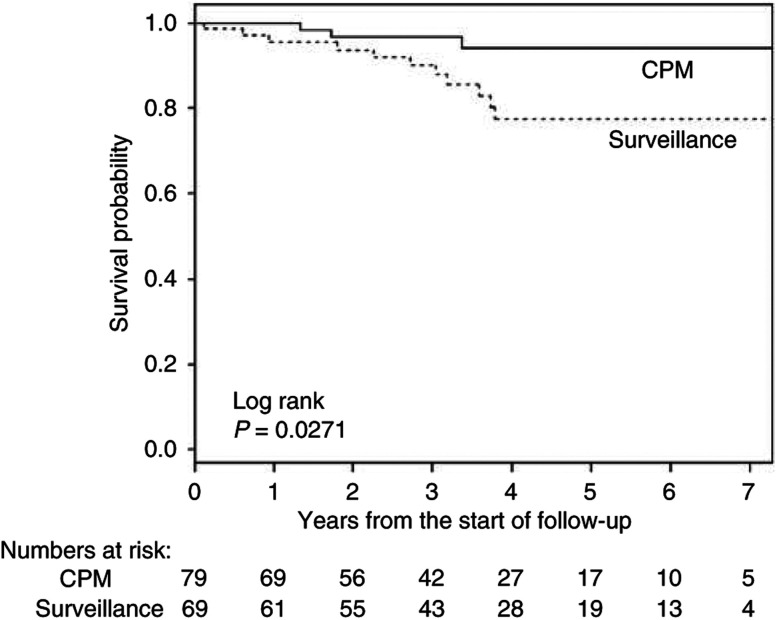
Overall survival in patients who opted for CPM *vs* patients who remained under surveillance.

In order to examine whether CPM prevented death from breast cancer, breast cancer-specific and overall survival were compared between the CPM and the surveillance group. The mean follow-up did not differ markedly between both groups, 3.4 *vs* 3.7 years, respectively (Table 3c). Most women in both groups died of breast cancer (Table 3a and c). Breast cancer-specific survival (including the first breast cancers) was not significantly better in the CPM group (log rank, *P*=0.11) (Figure 2). However, these were mostly related to the first breast cancer as only one of them (in the surveillance group) had developed a contralateral breast cancer. Therefore, it was not possible to evaluate contralateral breast cancer-specific survival. A significant overall survival advantage was observed in the CPM group compared to the surveillance group (log rank, *P*=0.027) (Figure 3) due to three additional events: two ovarian and one lung cancer. Multivariate Cox's proportional-hazards analysis showed that, after adjustment for BPO (and time between first breast cancer and start follow-up and chemotherapy treatment), women in the CPM group did not significantly have better survival than those under surveillance (overall mortality HR 0.35, *P*=0.14) (Table 4). Patients who underwent BPO had significantly better breast cancer specific (HR 0.15 (95% CI 0.04–0.51), *P*=0.003) and overall survival (HR 0.14 (95% CI 0.05–0.41), *P*<0.0001) than patients who did not undergo BPO. In multivariate analysis, with adjustment for CPM, time between first breast cancer between first breast cancer and start follow-up and chemotherapy, the breast cancer-specific survival was no longer significant (HR 0.28 (95% CI 0.07–1.11), *P*=0.07), while the impact of BPO on overall survival remained (HR 0.23 (95% CI 0.07–0.78), *P*=0.018). Having opted for both CPM and a BPO resulted in a significantly better survival than surveillance only (overall mortality HR 0.12 (0.03–0.46) and breast cancer-specific mortality HR 0.16 (0.04–0.61), both adjusted for time between first breast cancer and start follow-up and chemotherapy).

## DISCUSSION

Women with a *BRCA1* or *BRCA2* mutation and a personal history of breast cancer have high risks of developing contralateral breast cancer. In this study, CPM reduced the risk for contralateral breast cancer in *BRCA1* or *BRCA2* mutation carriers by 91%, independent of the impact of BPO. Unadjusted overall survival was better for the CPM group *vs* the surveillance group, but this was unexpectedly mostly due to the first breast cancer or other cancer-related events. In addition, after adjustment for BPO in a multivariate Cox analysis, the CPM effect was no longer significant.

The observed impact of CPM on contralateral breast cancer reduction is in agreement with studies on familial breast cancer. McDonnell *et al* (2001) studied the efficacy of CPM in 745 women, of whom 388 were premenopausal. They estimated a 90% reduction after a median follow-up, from the date of CPM, of 10 years by using the Anderson model (Anderson and Badzioch, 1985) to predict contralateral breast cancer risk. Peralta *et al* (2000) reported three contralateral breast cancers at the time of prophylactic mastectomy in 64 women who underwent a CPM. No other cases of contralateral breast cancer were observed after a median follow-up, from the date of first breast cancer, of 6.2 years, whereas 36 contralateral breast cancers occurred among 182 age-matched controls after a median follow-up of 6.8 years. Despite a significant improved disease-free survival (defined as the time to any breast cancer event, whether recurrence or second primary), these authors also could not observe an improved overall survival, even though neither study adjusted for BPO status.

Bilateral prophylactic oophorectomy reduces the risk of first breast cancer by approximately 50% and the risk of ovarian cancer by almost 95% (Kauff *et al*, 2002; Rebbeck *et al*, 2002). In our study, more women in the CPM group than in the surveillance group had undergone BPO. Bilateral prophylactic oophorectomy proved not to be a confounder in the contralateral breast cancer risk analysis (Table 4); however, it was a strong confounder in the survival analysis and clearly contributed to the difference in survival between the CPM and surveillance group. In addition, the impact of having opted for both CPM and a BPO *vs* surveillance resulted in a larger survival benefit than having opted for CPM or BPO alone.

Retrospective cohort studies considering breast cancer survival in *BRCA1* or *BRCA2* mutation carriers may suffer from ascertainment and testing bias. An ideal study design to evaluate the efficacy of CPM would be a prospective, randomised clinical trial, but such a trial is not feasible due to obvious ethical considerations. In this retrospective study, we tried to avoid overestimation of impact of the prophylactic procedure by testing and survival bias, by starting the follow-up at the date of positive DNA test result or date of CPM. By doing this, we excluded women from the surveillance group who were triggered to be tested by their diagnosis of contralateral breast cancer, precluding an overestimation of impact of the CPM (Klaren *et al*, 2003).

In the survival analyses, all patients were included, also the possibly oversampled cases with contralateral breast cancer, because they were still at risk at the date of testing. Their inclusion might have resulted in a spuriously elevated breast cancer-specific mortality. In our study, only two women who developed contralateral breast cancer in the surveillance group died so far. Therefore, we did not show an impact of CPM on survival in the total cohort nor in the smaller cohort (*n*=118) (overall survival in CPM *vs* surveillance: 94 *vs* 68%, *P*=0.0072; adjusted HR 0.31 (95% CI, 0.07–1.38)).

Despite the bias adjustment described, some differences between the groups remained: the CPM patients tended to come from families with a more severe familial breast cancer history (data not shown), were tested at a younger age and were tested more often close to their first breast cancer diagnosis. The latter can partly be logically explained because at average their breast cancer diagnosis was in a later calendar period when testing was more widely available already. In addition, patients coming from a family with a more severe breast cancer history may be more eager to get tested. However, these differences would lead to a higher background risk for contralateral breast cancer and worse survival in the CPM group and therefore might only strengthen our findings.

Even though stage did not differ between the groups, the CPM group seemingly had chosen, besides more BPO, for more aggressive therapy (chemotherapy, hormonal therapy and mastectomy) for their first breast cancer compared to the surveillance group. This difference in BPO and therapy might partly be explained by the fact that the patients in the CPM group were diagnosed in more recent calendar years and may partly be on initiative of women themselves, who may have higher fear of breast cancer because of their family history. The higher uptake of bilateral mastectomy by women with a positive family history has been supported by a study within a group of breast cancer patients with an uninformative BRCA test result (Schwartz *et al*, 2004). Although in our study women who opted for CPM had more often undergone ipsilateral mastectomy than those in the surveillance group (73 *vs* 41%), this did not have an impact on breast cancer-specific or overall survival (data not shown). Adjuvant chemotherapy has been associated with a decreased rate of contralateral breast cancer for breast cancer patients in general (Early Breast Cancer Trialists' Collaborative Group, 2001). In our study, however, neither chemotherapy nor mastectomy had confounding effects in the relation of CPM with contralateral breast cancer incidence. The low participation of tamoxifen use by the women in our study might be explained by their relatively young age as well as the year of their first breast cancer diagnosis, because the tamoxifen trials were mostly directed to postmenopausal women and the results published after the date at first breast cancer diagnosis in this population.

In summary, women with a *BRCA1* or *BRCA2* gene mutation and a history of unilateral invasive breast cancer are at increased risk for developing a primary contralateral breast cancer. This study shows that CPM significantly reduces the risk for contralateral cancer among *BRCA1* or *BRCA2* mutation carriers. In addition, after 5-year follow up, overall survival was better for the CPM group *vs* the surveillance group (*P*=0.03), but unexpectedly this was mainly due to the first breast cancer or other cancer-related events. After adjustment for BPO in a multivariate Cox analysis, the CPM effect was no longer significant. The choice for CPM is highly correlated with that for BPO, while only BPO leads to a significant improvement in overall survival so far. We do expect, considering the numbers of women with contralateral breast cancer in the surveillance group, that with longer follow-up CPM will be shown to improve contralateral breast cancer-specific survival. The choice for CPM must be seen within the context of other preventive measures in survival, such as BPO, tamoxifen use (Narod *et al*, 2000) and close surveillance.

## Figures and Tables

**Table 1:** Clinicopathological characteristics of first breast cancer for women who opted for CPM and women under surveillance^a^

**Characteristic**	**CPM group (*N*=79)**	**Surveillance group (*N*=69)**	***P*-value**
*Age at first breast cancer (years)*			
Mean±s.e.	38.0±0.9	39.4±1.0	0.295
Range	26–56	25–64	
			
*Year of diagnosis*			
Mean	1995	1991	<0.001
Median	1997	1993	
Range	1982–2002	1972–2002	
			
*Breast cancer stage*			
Stage I	32 (40.5)	25 (36.2)	0.324
Stage IIa	31 (39.2)	27 (39.1)	
Stage IIb	10 (12.7)	15 (21.7)	
Stage IIIa	6 (7.6)	2 (2.9)	
			
*Pathological T stage (cm)*			
T1: ⩽2	43 (54.4)	38 (55.1)	0.677
T2: 2–5	33 (41.8)	30 (43.5)	
T3: >5	3 (3.8)	1 (1.4)	
			
*Axillary status*			
Node negative	52 (65.8)	40 (58.0)	0.495
Node positive	27 (34.2)	29 (42.0)	
			
*Bloom & Richardson grade*			
Grade I	— (0.0)	1 (2.1)	0.481
Grade II	22 (32.4)	16 (33.3)	
Grade III	46 (67.6)	31 (64.6)	
			
*Morphology*			
Ductal	68 (89.5)	49 (75.4)	0.042
Other	8 (11.5)	16 (24.6)	
			
*Estrogen receptor status*			
Positive	15 (26.3)	17 (44.7)	0.078
Negative	42 (73.7)	21 (55.3)	
			
*Progesterone receptor status*			
Positive	12 (22.6)	12 (33.3)	0.332
Negative	41 (77.4)	24 (66.7)	
			
*Therapy*			
Breast-conserving surgery	21 (26.6)	41 (59.4)	<0.001
Radiotherapy	46 (58.2)	58 (84.1)	0.001
Chemotherapy	46 (58.2)	26 (37.7)	0.014
Hormonal therapy	15 (19.0)	4 (5.8)	0.025

CPM=contralateral prophylactic mastectomy; s.e.=standard error of the mean.

a Values represent *N* (%), unless stated otherwise.

**Table 2:** Characteristics of women who opted for CPM and women under surveillance

**Characteristic**	**CPM group (*N*=79)**	**Surveillance group (*N*=69)**	***P*-value**
*Mutation status, N (%)*			0.693
*BRCA1*	60 (75.9)	55 (79.7)	
*BRCA2*	19 (24.1)	14 (20.3)	
			
*Age at mutation analysis (years)*			
Mean±s.e.	41.5±0.9	46.7±1.1	<0.001
Range	27–61	26–76	
			
*Age at CPM (years)*			
Mean±s.e.	41.9±0.9	NA	
Range	27–61	NA	
			
*Bilateral (salpingo-)oophorectomy, N (%)*	63 (79.7)	46 (66.7)	0.092
Indication			
Prophylactic	61 (96.8)	39 (84.8)	0.009^a^
Ovarian carcinoma	2 (3.2)	4 (8.7)	
Metastasis breast cancer in ovary	— (0.0)	1 (2.2)	
Adjuvant treatment first breast cancer	— (0.0)	1 (2.2)	
Radiologic castration	— (0.0)	1 (2.2)	
			
*Age at bilateral prophylactic (salpingo-)oophorectomy (years)*			
Mean±s.e.	43.3±0.8	47.1±1.2	0.009
Range	30–60	35–64	
			
Ipsilateral mastectomy (before or at the start of follow-up)	79 (100)	34 (49.3)	<0.001

CPM=contralateral prophylactic mastectomy; s.e.=standard error of the mean; NA=not applicable.

a Comparing prophylactic only.

**Table 3a:** Outcome after first breast cancer in all patients in the CPM and surveillance group

**Outcome**	**CPM group (*N*=79)**	**Surveillance group (*N*=69)**	***P*-value**
Follow up after first breast cancer mean±s.e. (years)	7.4±0.5	10.5±0.7	0.035
			
*Contralateral breast cancer*			
*N* (%)	1^a^ (1.3)	32 (46.4)	<0.001
Time until occurrence (years)	5.4	6.4±0.8	
			
*Vital status, N (%)*			
Alive, disease free	73 (92.4)	50 (72.5)	
Alive, breast cancer	1 (1.3)	7 (10.1)	
Alive, ovarian cancer	2 (2.5)	1 (1.4)	
Deceased from breast cancer	3 (3.8)	8 (11.6)	
Deceased from ovarian cancer	— (0.0)	2 (2.9)	
Deceased from other cancer	— (0.0)	1 (1.4)	
			
*Recurrence, N (%)*			
Local recurrence	8 (10.1)	12 (17.4)	0.233
Regional recurrence	1 (1.3)	1 (1.4)	1.000
Distant metastasis	5 (6.3)	9 (13.0)	0.260
			
Interval between primary breast carcinoma and start of follow-up (Tables 3b and c)	4.0±0.5	6.8±0.7	0.001

CPM=contralateral prophylactic mastectomy; s.e.=standard error of the mean.

a Three ductal carcinoma *in situ* and one invasive breast cancers found after histological examination in the CPM were excluded.

**Table 3b:** Contralateral breast cancer incidence after the start of follow-up, which is at the date of mutation testing or date of CPM

**Outcome and follow-up**	**CPM group (*N*=75)**	**Surveillance group (*N*=43)**	***P*-value**
*Follow-up mean±s.e. (years)*			
Until contralateral breast cancer, death or end of follow-up	3.4±0.2	3.1±0.3	0.440
			
*Contralateral breast cancer*			
*N* (%)	1^a^ (1.3)	6^b^ (14)	0.009
Time until occurrence (years)	1.6	2.2±0.8	

CPM=contralateral prophylactic mastectomy; s.e.=standard error of the mean.

a Three ductal carcinoma *in situ* and one invasive breast cancers found after histological examination in the CPM were excluded.

b Contralateral breast cancers that occurred before the date of mutation testing (*n*=26) were excluded.

**Table 4:** Risk of contralateral breast cancer and overall survival of women under surveillance compared to women who opted for CPM

	**Univariate**			**Multivariate**		
	**HR**	**95% CI**	***P*-value**	**HR^a^**	**95% CI**	***P*-value**
*Risk of contralateral breast cancer* b						
CPM *vs* surveillance	0.09	0.01–0.78	0.03			
						
*Overall mortality* c						
CPM versus surveillance	0.26	0.07–0.94	0.04	0.35^d^	0.09–1.39	0.14

CPM=contralateral prophylactic mastectomy; s.e.=standard error of the mean; HR=hazard ratio; CI=confidence interval.

a HR derived from Cox's proportional-hazards analysis. Confounders (see Statistical analysis) were included if they changed the HR estimate by more than 10%.

b *N*=118 patients.

c *N*=148 patients.

d Adjusted for BPO, time between first breast cancer and the start of follow-up, and chemotherapy.

**Table 3c:** Vital status after the start of follow-up, which is at the date of mutation testing or the date of CPM

**Outcome and follow**-**up**	**CPM group (*N*=79)**	**Surveillance group (*N*=69)**	***P*-value**
*Follow-up mean±s.e. (years)*			
Until death or end of follow-up	3.4±0.2	3.7±0.2	0.337
			
*Vital status, N (%) (sum of Table 3a)*			0.021
Alive	76 (96.2)	58 (84.1)	
Deceased	3 (3.8)	11 (15.9)	

CPM=contralateral prophylactic mastectomy; s.e.=standard error of the mean.
